# Genetic Performance and General Combining Ability of Oil Palm Deli *dura* x AVROS *pisifera* Tested on Inland Soils

**DOI:** 10.1100/2012/792601

**Published:** 2012-05-22

**Authors:** A. Noh, M. Y. Rafii, G. Saleh, A. Kushairi, M. A. Latif

**Affiliations:** ^1^Malaysian Palm Oil Board, P.O. Box 10620, 50720 Kuala Lumpur, Malaysia; ^2^Department of Crop Science, Faculty of Agriculture, Universiti Putra Malaysia, 43400 UPM Serdang, Selangor, Malaysia; ^3^Institute of Tropical Agriculture (ITA), Universiti Putra Malaysia, 43400 UPM Serdang, Selangor, Malaysia

## Abstract

The performance of 11 oil palm AVROS (*Algemene Vereniging van Rubberplanters ter Oostkust van Sumatra*) *pisiferas* was evaluated based on their 40 *dura* x *pisifera* (DxP) progenies tested on inland soils, predominantly of Serdang Series. Fresh fruit bunch (FFB) yield of each *pisiferas* ranged from 121.93 to 143.9 kg palm^−1^ yr^−1^ with trial mean of 131.62 kg palm^−1^ yr^−1^. Analysis of variance (ANOVA) showed low genetic variability among *pisifera parents* for most of the characters indicating uniformity of the *pisifera* population. This was anticipated as the AVROS *pisiferas* were derived from small population and were inbred materials. However, some of the *pisiferas* have shown good general combining ability (GCA) for certain important economic traits. Three *pisiferas* (P1 (0.174/247), P3 (0.174/498), P11 (0.182/308)) were identified of having good GCA for FFB yield while *pisiferas* P1 (0.174/247), P10 (0.182/348), and P11 (0.182/308) were good combiners for oil-to-bunch ratio (O/B). The narrow genetic base of these materials was the main obstacle in breeding and population improvement. However, efforts have been made to introgress this material with the vast oil palm germplasm collections of MPOB for rectifying the problem.

## 1. Introduction

Palm oil is one of the world's healthiest oils. As a natural vegetable oil, it contains no transfatty acids or cholesterol. It is currently being used by doctors and government agencies to treat specific illnesses and improve nutritional status. Recent medical studies have shown that palm oil, particularly virgin (red) palm oil, can protect against many common health problems [1]. The history of AVROS oil palm *pisifera* in Malaysia begins with the importation of oil palm *tenera* x *pisifera* (TxP) seeds from Indonesia by the Department of Agriculture Malaysia and Harrisons and Crosfield and planted at Klanang Baru Estate in 1957 [2–4]. Progenies of this material were later planted in Trial 0.79 at Federal Experimental Station, Serdang, in 1965; later the *tenera *x *tenera* (TxT) and TxP of the material were planted at Malaysian Palm Oil Board (MPOB) Kluang in 1981 and 1982. Ever since, the AVROS *pisiferas* form the basic materials for MPOB research and commercial seed production. The AVROS *pisifera* progenies exhibit a high mesocarp-to-fruit and oil to bunch ratios but are tall [4–8].

The performance of AVROS *pisifera* as male parent in the DxP seed production had been tested by various agencies [9–15]. MPOB examined 27 Deli *dura* x AVROS progenies planted on coastal soil at 136 palms ha^−1^ in 1978 [16]. The average fresh fruit bunch (FFB) yield of the material over 15 years (1981–1995) was 23.81 t ha^−1^ yr^−1^ and the best progeny yielded 28.81 t ha^−1^ yr^−1^. The trial means for oil and kernel yields were 6.29 t ha^−1^ yr^−1^ and 1.53 t ha^−1^ yr^−1^, respectively. FELDA also evaluated *dura* x *dura *(DxD) and *dura* x *tenera *(DxT) progenies using Deli duras crossed with Yangambi, La Me', AVROS, NIFOR, and fertile *pisiferas *on inland soils [17, 18]. The FFB yields of the group of progenies were 22 to 25 t ha^−1^ yr^−1^ and extraction rate of 18.3% to 23.6%. Golden Hope also evaluated Deli *dura* x AVROS* pisifera* progenies in their commercial block for 15 years (1980–1994) [19]. The average FFB yields over 8–12 year production on coastal and inland estates were 28–30 t ha^−1^ yr^−1^ and 22–28 t ha^−1^ yr^−1^, respectively. In Oil Palm Research Station Dami, Papua New Guinea, the first four years FFB yield ranged from 16 to 29.7 t ha^−1^ yr^−1^ and oil to bunch (O/B) was 25.4% [19]. The performance of Deli *dura* x AVROS *pisifera *was also encouraging in ASD, Coto, Costa Rica [20]. The FFB yields were 12–18 t ha^−1^ yr^−1^ at Coto and 14–23 t ha^−1^ yr^−1^ at Palmar.

The AVROS *pisiferas* were known to have high general combining ability (GCA) [16]. Information on combining ability is essential to identify superior parents for hybrid seeds production. There are two types of combining abilities, general combining ability (GCA) and specific combining ability (SCA). GCA is a useful to identify parents for the development of superior genotypes while SCA for providing information about the performance of hybrids [21]. The differences in GCA are mainly due to the additive genetic effects while differences in SCA are attributed to the nonadditive dominance and other types of epistasis [22]. In oil palm, studies by Breure and Konimor [23] in Deli-AVROS population reported that exploiting GCA and SCA among parents could increase fresh fruit bunch (FFB) yield, oil-to-bunch ratio (O/B), and kernel-to-bunch ratio (K/B) by 42%, 18%, and 29%, respectively. Dumortier and Konimor [24] suggested that AVROS *pisifera,* progeny DM 742, had good GCA for FFB yield, bunch number per palm (BNO), O/B, and leaf area but had low GCA for frond dry weight. Musa [25] reported that AVROS male parents MS 218/24, MS 2182/16, and MS 2193/55 were good general combiners with Deli *dura* for FFB yield (8.35, 6.24, and 22.74 kg palm^−1^ yr^−1^, resp.) and BNO (1.89, 1.99, and 2.86 bunches palm^−1^, resp.). The male parents MS 2188/85, MS 2182/24, MS 2182/16 and MS2186/67 had good GCA for O/B (0.59,0.69, 0.96, and 0.46%, resp.). This paper highlights the performance of some MPOB'S AVROS *pisiferas *and their general combining ability (GCA) planted on inland soils of predominantly Serdang Series.

## 2. Material and Method

A total of 40 progenies of oil palm Deli *dura* x AVROS* pisifera* (DxP) were planted in trial 0.314 at MPOB Keratong Station in 1994. The Deli *dura* materials originated from the Sabah Breeding Programme (SPB) were used as female parents. The male parents were the AVROS *pisiferas*, the descendants of BM 119 from Oil Palm Research Station, Banting, Selangor. The materials were crossed using North Carolina Mating Design I (NCM I) [26]. NCM I is a nested design, where every male is mated to a number of females in a set. It can be used to estimate genetic variance components that is additive and dominance variances and narrow-sense heritability. The progenies were created by randomly crossing each of the 11 male *pisiferas *with sets of two to six female* duras*. The *pisifera *palms were identified as “P” (P1–P11). Mean annual rainfall (1993–2004) was 2051.44 mm per year with the range from 984 mm to 3314 mm per year. Data collections were carried out for bunch yield (1998–2004), bunch quality components (1999–2004), and one round vegetative measurement (2003). 

### 2.1. Data Collection and Statistical Analysis

Data on the following component characters were collected. The bunch yield components were fresh fruit bunch (FFB), bunch number (BNO), and average bunch weight (ABW). The bunch quality components included fruit to bunch (F/B), oil to bunch (O/B), kernel to bunch (K/B), mesocarp-to-fruit (M/F), shell to fruit (S/F), oil to dry mesocarp (O/DM), oil yield (OY), kernel yield (KY), and total economic product (TEP) while vegetative traits included frond production (FP), petiole cross section (PCS), rachis length (RL), leaflet length (LL), leaflet width (LW), leaflet number (LN), palm height (HT), leaf area (LA), leaf area index (LAI), and diameter (Dia). 

The data collection was based on individual palm basis and was computed using the Statistical Analysis System (SAS) program. Simple statistics for each trait such as Mean, Standard Error (SE), and Standard Deviation (SD) were determined. Analyses of variance (ANOVA) among traits also were carried out by SAS program. Duncan New Multiple Range Test (DNMRT) tests for progenies means comparison. 

### 2.2. General Combining Ability (GCA) Estimates

The GCA values of the parents were estimated using the method introduced by Kempthorne [27] as stated hereinafter:

(1) $$\begin{array}{c} G_{i}\;=\;\frac{X_{i}}{n_{1}}-\frac{X}{n_{2}}\cdots ,\;\\ \text{with}\;\text{SE}\;=\;\frac{\sqrt{\text{M}1}}{rf\;}\; \end{array}$$

where, *G*_*i*_ is the GCA value for the **i**th male; *X*_*i*_ is the Total value for the *i*th male;  *X*…  is the grand total, *n*_1_ and *n*_2_ are the number of observation on *n*_1_ and *n*_2_, respectively; SE is the standard error; *M*1 is the means squares of error; *r* is the number of replications; *d* is the number of *dura/pisifera.*

## 3. Result and Discussion

### 3.1. Yield and Yield Components

The ANOVA for yield and its components is presented in Table 1. Among *pisifera *male parents were showed nonsignificant differences for fresh fruit bunch (FFB) yield, bunch number per palm (BNO), and average bunch weight (ABW). The replicates by *pisiferas* interaction were nonsignificant for FFB and ABW but highly significant for BNO. The result indicated the consistencies in performance of the *pisifera* male parents for the FFB and ABW across the replicates but not for BNO. Effects of *duras* within *pisifera* were shown significant different for BNO but not for FFB and ABW traits. Interaction effects between replicates and *duras*-within-*pisifera* were significant for FFB yield and yield components, implying that the *dura *female parents-within-*pisifera* male parent differ in their performance across the replicates. 

Yield and yield components based on male parents are presented in Table 2. Mean performance of the *pisiferas* showed that the grand mean for FFB was 131.62 kg palm^−1^ yr^−1^, with mean BNO of 8.66 bunches palm^−1^ yr^−1^ and average bunch weight (ABW) of 15.60 kg bunch^−1^. Among the eleven *pisiferas*, P1 (0.174/247) gave the highest *tenera *FFB yield of 143.90 kg palm^−1 ^yr^−1^, more than 9% above the grand mean. The high FFB yield was attributed to the high and balanced BNO (9.06 bunches palm^−1^ yr^−1^) and ABW (16.25 kg bunch^−1^). High FFB yields were also observed in P3 (0.174/498) (143.50 kg palm^−1^ yr^−1^) and P11 (0.182/308) (143.37 kg palm^−1^ yr^−1^). Conversely, P6 experienced low FFB yields due to its lowest ABW (14.40 kg bunch^−1^) and below average BNO (8.53 bunches palm^−1^ yr^−1^). Duncan New Multiple Range Test (DNMRT) also indicated significant differences between P6 and P1, P3, P11 for FFB, BNO, and ABW. The result also indicated that P2 (0.174/348) even though with the highest BNO (9.46 bunches palm^−1^ yr^−1^), failed to register among the top FFB yielder due to its low ABW (14.93 kg bunch^−1^). Similarly, P9 (0.182/297) with the highest ABW (16.77 kg bunch^−1^) showed below average FFB yield (131.11 kg palm^−1^ yr^−1^) due to poor BNO (8.08 bunches palm^−1 ^yr^−1^). It is therefore important in selection, to select palms with high BNO and moderate ABW for high FFB yield. 

General combining ability (GCA) estimates for bunch yield and yield components are presented in Table 3. The results indicated that the male parents P1 (0.174/247), P3 (0.174/498) and P11 (0.182/308) were good general combiners for FFB and its components. Their GCA values for P1 (0.174/247) for FFB, BNO and ABW were 12.28 kg palm^−1^ yr^−1^, 0.40 bunches palm^−1^ yr^−1^and 0.65 kg bunch^−1^, respectively. For P3 (0.174/498) their GCA values were FFB 11.88 kg palm^−1^ yr^−1^, BNO 0.37 bunches palm^−1^ yr^−1^, and ABW 0.71 kg bunch^−1^and GCA estimates for P11 (0.182/308) were FFB 11.75 kg palm^−1^  yr^−1^, BNO 0.78 bunches, and ABW 0.23 kg. Among the eleven *pisiferas*, the best general combiner for FFB was P1(0.174/247), P2 (0.174/348) for BNO, and P7 (0.182/77) for ABW. Dumortier and Konimor [24] noted that the AVROS *pisifera* of DM 742 had good GCA for FFB yield and bunch number (BNO). 

### 3.2. Bunch Quality Components

The ANOVA for bunch quality components is presented in Table 4. Among *pisifera *male parents were shown to have significant difference for mesocarp fruit weight (MFW), kernel-to-bunch ratio (K/B), kernel yield (KY) and to be highly significant for fruit-to-bunch ratio (F/B). The interaction effects between replicates x *pisifera *male parents were found to be highly significant for MFW, mean nut weight (MNW), mesocarp-to-fruit ratio (M/F), shell-to-fruit ratio (S/F), oil-to-dry mesocarp ratio (O/DM), oil yield (OY), and KY. The *dura* females within *pisifera* males were, however, highly significant for all the bunch quality traits except F/B. The interaction effects of replicates by females-within-male were also highly significant except O/DM, indicating the differences in behavior of the *dura* females within male in the three replicates. 

The performances of the 11 *pisifera* male parents are presented in Table 5. The *pisifera* P11 (0.182/308) and P10 had good fruit-to-bunch ratio (F/B) with 66.77% and 66.54%, respectively. DNMRT indicated that they differed significantly from P5 (53.21%), which had the lowest F/B ratio. The two *pisifera*s also exhibited the highest oil-to-bunch ratio (O/B) among the *pisiferas* with 26.23% for P11 and 26.50% for P10. A total of four male parents, P3, P1, P8, and P11, had kernel-to-bunch ratio (K/B) of more than 6%, which is higher than or more than that from the trial mean of 5%. DNMRT indicated, that they differed significantly with the other *pisiferas* used in the trial. The *pisifera* P10 had mean fruit weight ratio (MFW) of 12.44 g, the highest among the male parent. DNMRT detected significant differences from other male parents. Four male parents, P5, P2, P8, P11, and P10, had mesocarp-to-fruit ratio (M/F) of more than 80%. DNMRT indicated significant differences between them and the other male parents but no differences were detected between the four male parents. The *pisifera *P2 had the lowest shell-to-fruit ratio (S/F) with 9.23% and consequently the highest M/F with 80% among the male parents. Conversely, male parent P4 had the highest S/F (12.90%) and as a result the lowest M/F among the male parents. The oil-to-dry-mesocarp ratio (O/DM) and oil yield (OY) were derived characters and were reasonably good with the trial means of 78.76% and 31.15 kg/p/yr, respectively. The best male parent for O/DM, P7 (79.47%), did not differ significantly with the other except with the lowest male parent P8 (78.18%). Five male parents; P11, P1, P3, P10, and P2, were good oil yielder, above the trial mean of 31.15 kg palm^−1^ yr^−1^. DNMRT indicated, that they differ significantly with the other male parents but no differences were detected among them. For kernel yield (KY) and total economic product (TEP), P1 with 8.46 kg palm^−1^ yr^−1^ was the highest for KY and P11 with 40.02 kg palm^−1^ yr^−1^ was the highest for TEP (DNMRT indicated that they differ significantly with most the other male parents). 

The estimates of GCA for the 11 *pisiferas* are shown in Table 6. The *pisifera* male parent P10 (0.182/305) was the best general combiner for O/B (1.59%) and among the best combiners for F/B. P11 (0.182/308) was the best combiner for F/B (2.22%), oil yield (OY) (4.42 kg palm^−1^ yr^−1^) and total economic product (TEP) (4.63 kg palm^−1^ yr^−1^). Three *pisiferas* (P3 (0.174/498), P1 (0.174/247) and P7 (0.182/77)) showed good GCA for K/B. P1 was also a good combiner for OY, KY, and TEP. In the M/F, P5 (2.21%) and P2 (2.07%) showed good combiners for the character. For S/F, P4 (0.174/663) (2.08%) and P7 (0.82/77) (1.89%) were good combiners. The best GCA for TEP was P11 followed by P1 and P3. 

### 3.3. Vegetative Traits

The ANOVA for the vegetative characters is shown in Table 7. Among the *pisifera *male parents were shown highly significant difference for leaflet length (LL) and significant difference for leaf area (LA) and leaf area index (LAI) but not significance for the other traits, indicating the substantial variation still existed in LL, LA, and LAI among *pisiferas*. Interaction between replicates and *pisifera* males was highly significant for all the vegetative traits, suggesting inconsistent behavior of the *pisifera* male parents across the replicates for those traits. Unlike among *pisifera *male parents, the *dura* females within *pisifera* male exhibited highly significant difference for all the vegetative traits. The result indicated that substantial variation still exist in the *dura* females within *pisifera *males and can be utilized for further selection and improvement. The replicates by *dura *females-within-*pisifera *male item were also highly significant, implying the differences in performance of the *dura *females-within-*pisifera *male in the three replicates. 

Mean performance of the progenies for vegetative characters pooled over *pisifera *male parents is presented in Table 8. Short trunk height (HT) and smaller trunk diameter (DIA) are preferred since they may prolong economic life and more nutrient can be channeled in FFB production instead of vegetative growth and maintenance. The *pisifera* P1 (0.174/247) and P8 (0.182/230) had the lowest DIA of 0.61 m. P6 (0.182/30) and P4 (0.174/663) registered the shortest HT of 2.28 m and 2.29 m, respectively. DNMRT indicated significant differences between P1 (0.174/247) and P8 (0.182/230) with most of the *pisiferas* for HT. Likewise P6 (0.182/30) and P8 (0.182/230) exhibited significant differences for DIA with all male parents except for P6 (0.182/30) and P9 (0.182/297). 

In oil palm, the inflorescence is embedded at the frond axil; frond production rate determines the limit to bunch production. Each frond subtends only one inflorescence that can be potentially male or female. Besides low HT, P4 exhibited highest frond production (FP) of 27.63 fronds yr^−1^, equivalent to 2.3 fronds month^−1^. Male parent P1 also had high FP of 27.09 fronds yr^−1^and lowest DIA among the male parents. DNMRT indicated that the two *pisiferas* were significantly different with majority of the male parents. *Pisifera* P8 (0.182/230) had the lowest petiole cross section (PCS), 26.61 cm^2^, and rachis length (RL), 5.35 m. Palms with low PCS and RL values are preferred in breeding and selection since they may increase the planting density per hectare. DNMRT indicated significant differences between *pisifera *P8 and most of the male parents. 

Leaflet length (LL) and leaflet width (LW) based on male parents ranged from 93.57 to 101.07 cm and 5.37 to 5.90 cm, respectively. P10 (0.182/305) had the highest LL and LW among the male parents, while *pisifera *P11 (0.182/308) registered with the shortest LL and *pisifera *P8 had the lowest LW. For LL, DNMRT showed significant differences between *pisifera *P10 (0.182/305) and all the other *pisiferas* except *pisifera *P3 (0.174/498). DNMRT also detected significant differences for LW between *pisifera *P8 (0.182/230) and majority of the male parents. In this study, frond with the highest leaflet number (LN) was recorded from *pisifera *P11 (0.182/308) with 173.69 leaflets frond^−1^ and *pisifera *P6 (0.182/30) was the lowest with 163.67 leaflets frond^−1^. Leaf area (LA) is a derived character, with the components of LL, LW, and LN. Among the *pisiferas*, male parent *pisifera* P10 (0.182/305) had the highest LA with 11.48 cm^2^ and P8 (0.182/230) registered as the lowest with 9.12 cm^2^. DNMRT showed significant differences for LA between the two *pisiferas* and the other male parents. 

The GCA estimates of male parents for vegetative characters are presented in Table 9. In traits such as trunk height (HT), trunk diameter (DIA), rachis length (RL), and petiole cross section (PCS), negative values are preferred since they satisfies selection criteria of low values. The male parents *pisifera* P1 (0.174/247) and *pisifera* P8 (0.182/230) were good general combiners for low trunk diameter with both had GCA estimates of −0.02 m. The males P6, P4, and P9 had good GCA for lower trunk height, −0.16, −0.15 and −0.13 m, respectively. Male parents P8, P5, and P4 were good combiners for shorter rachis length with GCA values of −0.21, −0.19, and 0.13 m, respectively. P8 was the best combiner for smaller PCS (−2.41 cm^2^) followed by P9 with −2.19 cm^2^. Male parents with high GCA for LA were P10 (0.78 cm^2^), P3 (0.44 cm^2^), and P11 (0.34 cm^2^). Overall, P8 was a good combiners for low trunk diameter, low height, short RL and small PCS. Musa [25] in his studies on two Deli-AVROS populations found that two male parents (AVROS *pisifera*) (MS 2182/16 and MS 2188/97) had the capability to transmit low trunk height, low trunk girth and short rachis length, because of their high negative GCA for those traits. 

## 4. Conclusion

The performance of 11 oil palm AVROS *pisiferas* was evaluated in inland soils, predominantly of Serdang Series. Analysis of variance (ANOVA) showed low genetic variability among *pisifera parents *for most of the characters indicating uniformity of the *pisifera* population. This was anticipated as the AVROS *pisiferas* were derived from small population and were inbred materials. For male parent selection, general combining ability (GCA) may have to be considered. Three *pisiferas* (P1 (0.174/247), P3 (0.174/498), P11 (0.182/308)) were identified of having good GCA for FFB yield. For O/B, the good combiners were P1 (0.174/247), P10 (0.182/348) and P11 (0.182/308). The good combiners for vegetative traits were P6 (0.182/30), P8 (0.182/230), and P9 (0.182/297). They can be considered for a single trait or in combination with the other for their selection. For instance, P1 (0.174/247) and P11 (0.182/308) were good candidates in selecting *pisiferas* with good GCA for FFB yield and O/B but not for vegetative characters. *Pisiferas* P6 (0.182/30), P8 (0.182/230), P9 (0.182/297) have good GCA value for lower trunk height (HT), lower trunk diameter (DIA), small petiole cross section (PCS) and short rachis length (RL). They can be considered for the production of relatively less vigorous growing palms. 

## Figures and Tables

**Table 1:** Mean squares of yield and yield components.

Source of variation	df	FFB	BNO	ABW
Replications	2	49137.61	268.98	110.87
*Pisifera* males	10	7605.62ns	27.50ns	79.07ns
*Dura *females/*pisifera *male	29	5832.9ns	29.73*	64.83ns
Replications x *pisifera* males	20	4734.22ns	33.61**	21.32ns
Replications x *dura *females/*pisifera *male	58	4471.24**	15.79**	43.28**
Within palms	1224	898.36	4.88	9.89

*Significant at 5% level; **Significant at 1% level; ns: non-significant; df: degrees of freedom; FFB: fresh fruit bunch; BNO: bunch number; ABW: average bunch weight.

**Table 2:** Means of *dura *x* pisifera *with different *pisifera* male parents for yield and yield components.

* Pisifera *male	Yield and yield components		
	FFB (kg palm^−1^ yr^−1^)	BNO (bunches palm^−1^ yr^−1^)	ABW (kg bunch^−1^)
P1 (0.174/247)	143.90a	9.06ba	16.25bc
P2 (0.174/348)	136.00ba	9.46a	14.93de
P3 (0.174/498)	143.50a	9.03ba	16.31bc
P4 (0.174/663)	122.87ed	8.20d	15.25de
P5 (0.182/7)	126.23ecd	8.34dc	15.32d
P6 (0.182/30)	121.93e	8.53bdc	14.40e
P7 (0.182/77)	132.18bc	8.00d	17.27a
P8 (0.182/230)	123.47ed	8.34dc	15.19de
P9 (0.182/297)	131.11bcd	8.08d	16.77ba
P10 (0.182/305)	133.31bc	8.85bac	15.63dc
P11 (0.182/308)	143.37a	9.44a	15.83dc

Mean	131.62	8.66	15.6
Standard Error	4.74	0.35	0.50

FFB: fresh fruit bunch; BNO: bunch number; ABW: average bunch weight.

Lettering indicates the difference between treatments. Means with the same small letter(s) in the same column are not significantly different at *P* ≤ 0.05 with Duncan New Multiple Range Test (DNMRT).

**Table 3:** General combining ability (GCA) estimates for yield and yield components for *pisifera *male parents.

*Pisifera* male	FFB (kg palm^−1^ yr^−1^)	BNO (bunches palm^−1^ yr^−1^)	ABW (kg bunch^−1^)
P1 (0.174/247)	12.28	0.4	0.65
P2 (0.174/348)	4.38	0.8	−0.67
P3 (0.174/498)	11.88	0.37	0.71
P4 (0.174/663)	−8.75	−0.46	−0.35
P5 (0.182/7)	−5.39	−0.32	−0.28
P6 (0.182/30)	−9.69	−0.13	−1.2
P7 (0.182/77)	0.56	−0.66	1.67
P8 (0.182/230)	−8.15	−0.32	−0.41
P9 (0.182/297)	−0.51	−0.58	1.17
P10 (0.182/305)	1.69	0.19	0.03
P11 (0.182/308)	11.75	0.78	0.23

FFB: fresh fruit bunch; BNO: bunch number; ABW: average bunch weight.

**Table 4:** Mean squares of bunch quality characteristics.

Source of variation	df	F/B	O/B	K/B	M/F	S/F	O/DM	OY	KY	TEP
Replications	2	228.56	67.88	42.68	158.24	23.08	30.51	1568.76	301.58	2501.74
*Pisifera* males	10	289.85**	58.82ns	17.16*	219.01ns	99.49ns	0.94ns	422.35ns	42.31*	521.30ns
*Dura *females/*pisifera *male	29	73.73ns	10.42**	7.71**	117.73**	63.69**	17.87**	257.65**	19.49**	300.94**
Replications x *Pisifera* males	20	42.67 ns	14.35ns	1.75ns	33.81*	17.03**	14.30**	274.80**	10.87**	327.93**
Replications x *Dura *females/*pisifera *male	58	79.41**	25.66**	4.82**	45.74**	20.10**	14.30**	192.46**	15.12**	236.63**
Within palms	835	50.86	15.71	2.33	18.08	8.01	7.48	67.75	5.21	78.64

*Significant at 5% level; **Significant at 1% level; ns: non-significant; df: degrees of freedom; F/B: fruit-to-bunch ratio, O/B: oil-to-bunch ratio, K/B: kernel-to-bunch ratio; M/F: mesocarp-to-fruit ratio; S/F: shell to fruit ratio; O/DM: oil-to-dry mesocarp ratio; OY: oil yield; KY: kernel yield; TEP: total economic product.

**Table 5:** Means of *dura *x* pisifera *with different *pisifera* male parents for bunch quality characteristics.

* Pisifera *male	F/B (%)	O/B (%)	K/B (%)	M/F (%)	S/F (%)	O/DM (%)	OY (kg palm^−1^ yr^−1^)	KY (kg palm^−1^ yr^−1^)	TEP (kg palm^−1^ yr^−1^)
P1(0.174/247)	65.78ba	25.13bc	6.24a	79.07cb	11.45b	78.74ba	34.21ba	8.46a	39.28ba
P2 (0.174/348)	63.42bc	24.92bc	5.35cb	82.51a	9.23e	78.92ba	32.11bdc	6.79ced	36.18edc
P3 (0.174/498)	65.11bac	24.39dc	6.25a	79.01cb	11.46b	78.35b	33.02bac	8.40a	38.07bac
P4 (0.174/663)	65.32bac	24.87bc	5.98a	78.02c	12.90a	78.93ba	28.95ef	7.07cbd	33.19egf
P5 (0.182/7)	63.21c	24.94bc	4.86c	82.65a	9.67de	78.73ba	29.10ef	5.66f	32.50gf
P6 (0.182/30)	65.07bac	24.90bc	5.93a	79.98b	10.93cb	78.49ba	30.69edc	7.27cb	35.06edf
P7 (0.182/77)	64.78bac	24.02dc	6.23a	77.65c	12.71a	79.47a	29.81ed	7.65b	34.40edf
P8 (0.182/230)	60.14d	23.33d	5.27cb	81.80a	9.50e	78.18b	27.00f	6.11fe	30.66g
P9 (0.182/297)	63.79bc	24.12dc	6.07a	78.87cb	11.72b	78.97ba	29.61edf	7.23cbd	33.94edf
P10 (0.182/305)	66.54a	26.50a	5.21cb	81.67a	10.50cd	78.87ba	32.90bac	6.49ed	36.80bdc
P11 (0.182/308)	66.77a	26.23ba	5.46b	81.68a	10.14cde	79.03ba	35.57a	7.42cb	40.02a

Mean	64.55	24.91	5.67	80.44	10.82	78.76	31.15	7.07	35.39
Standard Error	1.13	0.63	0.24	0.67	6.32	0.43	1.3	0.36	1.4

F/B: fruit to bunch; O/B: oil to bunch; K/B: kernel to bunch; M/F: mesocarp-to-fruit; S/F: shell to fruit; O/DM: oil to dry mesocarp; OY: oil yield; KY: kernel yield; TEP: total economic product.

Lettering indicates the difference between treatments. Means with the same small letter(s) in the same column are not significantly different at *P* ≤ 0.05 with Duncan New Multiple Range Test (DNMRT).

**Table 6:** General combining ability (GCA) estimates for bunch quality characteristics for *pisifera* male parents.

* Pisifera *male	F/B (%)	O/B (%)	K/B (%)	M/F (%)	S/F (%)	O/DM (%)	OY (kg palm^−1^ yr^−1^)	KY (kg palm^−1 ^yr^−1^)	TEP (kg palm^−1^ yr^−1^)
P1 (0.174/247)	1.23	0.22	0.57	−1.37	0.63	−0.02	3.06	1.392	3.89
P2 (0.174/348)	−1.13	0.01	−0.32	2.07	−1.59	0.16	0.96	−0.28	0.79
P3 (0.174/498)	0.56	−0.52	0.58	−1.43	0.64	−0.41	1.87	1.33	2.68
P4 (0.174/663)	0.77	−0.04	0.31	−2.42	2.08	0.17	−2.2	0	−2.20
P5 (0.182/7)	−1.34	0.03	−0.81	2.21	−1.15	−0.03	−2.05	−1.41	−2.89
P6 (0.182/30)	0.52	−0.01	0.26	−0.46	0.11	−0.27	−0.46	0.2	−0.33
P7 (0.182/77)	0.23	−0.89	0.56	−2.79	1.89	0.71	−1.34	0.58	−0.99
P8 (0.182/230)	−4.41	−1.58	−0.4	1.36	−1.32	−0.58	−4.15	−0.96	−4.73
P9 (0.182/297)	−0.76	−0.79	0.4	−1.57	0.9	0.21	−1.54	0.16	−1.45
P10 (0.182/305)	1.99	1.59	−0.46	1.23	−0.32	0.11	1.75	−0.58	1.41
P11 (0.182/308)	2.22	1.32	−0.21	1.24	−0.68	0.27	4.42	0.35	4.63

F/B:  fruit to bunch; O/B: oil to bunch; K/B:  kernel to bunch; M/F: mesocarp-to-fruit; S/F: shell to fruit; O/DM: oil to dry mesocarp; OY: oil yield; KY:  kernel yield; TEP: total economic product.

**Table 7:** Mean squares of vegetative traits.

Source of variation	df	FP	PCS	RL	LL	LW	LN	HT	LA	LAI	DIA
Replications	2	11.52	1150.96	5.25	579.77	13.51	3621.24	0.89	111.48	39.06	0.01
*Pisifera* males	10	25.65 ns	491.60 ns	2.82 ns	1346.55**	2.22 ns	823.97 ns	2.17 ns	44.27*	15.51*	0.04 ns
*Dura *females/*pisifera m*ale	29	28.65**	289.19**	3.10**	372.41**	2.08**	735.50**	1.54**	15.99**	5.60**	0.05**
Replications x *Pisifera* males	20	18.39**	177.07**	0.75**	139.74**	1.39**	302.37**	1.05**	9.86**	3.45**	0.01**
Replications x *Dura *females/*pisifera m*ale	58	14.76**	211.40**	1.61**	184.06**	1.80**	511.70**	1.49**	15.94**	5.58**	0.01**
Within palms	1223	6.77	37.79	0.17	45.96	0.25	118.04	0.16	2.33	0.82	0.01

*Significant at 5% level; **Significant at 1% level; ns: non-significant; df: degrees of freedom.

FP: frond production; PCS: petiole cross section; RL:  rachis length; LL: leaflet length; LW: leaflet width, LN:  leaflet number; HT: palm height; LA: leaf area; LAI: leaf area index; DIA: diameter.

**Table 8:** Means of *dura *x* pisifera *with different *pisifera *male parents for vegetative traits.

* Pisifera *male	FP (fronds palm^−1^ yr^−1^)	PCS (cm^2^)	RL (cm)	LL (cm)	LW (cm)	LN (no)	HT (m)	LA (m^2^)	LAI	DIA (cm)
P1(0.174/247)	27.09ba	28.43cbd	5.57cbd	93.83d	5.56cd	168.45bc	2.60a	10.02ed	5.93ed	0.61f
P2 (0.174/348)	26.93bac	28.16cebd	5.54ced	94.54cd	5.54cd	166.76dc	2.42b	10.00ed	5.92ed	0.63dce
P3 (0.174/498)	26.73bdc	31.39a	5.91a	99.93a	5.61cbd	171.01ba	2.47b	10.92b	6.46b	0.67a
P4 (0.174/663)	27.63a	27.73cebd	5.43fe	95.28cbd	5.370e	167.63dc	2.29c	9.85ef	5.83ef	0.65b
P5 (0.182/7)	26.84bdc	29.17b	5.37f	90.92e	5.52cd	164.73de	2.43b	9.48gf	5.61gf	0.63dce
P6 (0.182/30)	26.33bedc	27.01ced	5.48ed	94.11cd	5.53cd	163.67e	2.28c	9.79ef	5.79ef	0.62dfe
P7 (0.182/77)	26.48bedc	28.72cb	5.60cb	96.75b	5.64cb	168.70bc	2.43b	10.57cb	6.26cb	0.63c
P8 (0.182/230)	25.92e	26.61e	5.35f	88.49f	5.37e	166.11dce	2.36cb	9.12g	5.40g	0.61f
P9 (0.182/297)	26.10ed	26.82ed	5.67b	95.98cb	5.48ed	171.78a	2.31c	10.33cd	6.11cd	0.62dfce
P10 (0.182/305)	26.20edc	32.83a	5.64cb	101.07a	5.90a	167.75dc	2.67a	11.48a	6.80a	0.63dce
P11 (0.182/308)	26.47bedc	32.18a	5.82a	93.57d	5.73b	173.69a	2.64a	10.74cb	6.36b	0.62fe

Mean	26.64	29.01	5.56	94.89	5.57	167.71	2.44	10.17	6.02	0.63
Standard Error	0.41	0.97	0.07	1.07	0.08	1.72	0.06	0.24	0.14	0.02

FP = frond production; PCS: petiole cross section; RL: rachis length; LL: leaflet length; LW: leaflet width; LN: leaflet number; HT: palm height; LA: leaf area; LAI: leaf area index; DIA: diameter.

Lettering indicates the difference between treatments. Means with the same small letter (s) in the same column are not significantly different at *P* ≤ 0.05 with Duncan New Multiple Range Test (DNMRT).

**Table 9:** General combining ability (GCA) estimates on vegetative traits for *pisifera* male parents.

* Pisifera *male	HT (m)	DIA (cm)	FP (no. p/yr)	PCS (cm^2^)	RL (cm)	LL (cm)	LW (cm)	LN (no/p/yr)	LA (m^2^)	LAI (cm)
P1 (0.174/247)	0.16	−0.02	0.45	−0.58	0.01	−1.06	−0.01	0.74	−0.15	−0.09
P2 (0.174/348)	−0.02	0	0.29	−0.85	−0.02	−0.35	−0.03	−0.95	−0.17	5.78
P3 (0.174/498)	0.03	0.04	0.09	2.38	0.35	5.04	0.04	3.3	0.75	6.46
P4 (0.174/663)	−0.15	0.02	0.99	−1.28	−0.13	0.39	−0.2	−0.08	−0.32	5.83
P5 (0.182/7)	−0.01	0	0.2	0.16	−0.19	−3.97	−0.05	−2.98	−0.69	5.61
P6 (0.182/30)	−0.16	−0.01	−0.31	−2	−0.08	−0.78	−0.04	−4.04	−0.38	5.79
P7 (0.182/77)	−0.01	0	−0.16	−0.29	0.04	1.86	0.07	0.99	0.4	6.26
P8 (0.182/230)	−0.08	−0.02	−0.72	−2.4	−0.21	−6.4	−0.2	−1.6	−1.05	5.4
P9 (0.182/297)	−0.13	−0.01	−0.54	−2.19	0.11	1.09	−0.09	4.07	0.16	6.11
P10 (0.182/305)	0.23	0	−0.44	3.82	0.08	6.18	0.33	0.04	1.31	6.8
P11 (0.182/308)	0.2	−0.01	−0.17	3.17	0.26	−1.32	0.16	5.98	0.57	6.36

HT: palm height; DIA: diameter; FP: frond production; PCS: petiole cross section; RL: rachis length; LL: leaflet length; LW: leaflet width; LN: leaflet number; LA: Leaf Area; LAI: Leaf Area Index.
